# Effects of High-Fructose Corn Syrup on Bone Health and Gastrointestinal Microbiota in Growing Male Mice

**DOI:** 10.3389/fnut.2022.829396

**Published:** 2022-03-30

**Authors:** Xiaoqiang Han, Zhiguo Feng, Yizhang Chen, Liying Zhu, Xiaoqiong Li, Xin Wang, Haibiao Sun, Jinjun Li

**Affiliations:** ^1^Department of Orthopedics, The First Hospital of Shanxi Medical University, Taiyuan, China; ^2^First Clinical Medical College, Shanxi Medical University, Taiyuan, China; ^3^Institute of Food Science, Zhejiang Academy of Agricultural Sciences, Hangzhou, China; ^4^State Key Laboratory for Managing Biotic and Chemical Threats to the Quality and Safety of Agro-Products, Zhejiang Academy of Agricultural Sciences, Hangzhou, China

**Keywords:** high-fructose corn syrup, bone health, bone metabolism, gastrointestinal microbiota, functional nucleic acids

## Abstract

Here, we explored the correlation between gut microbiota and bone health and the effects of high-fructose corn syrup (HFCS) on both. Sixteen 3-week-old male C57BL/6J mice were randomly divided into two groups and given purified water (control group) or 30% HFCS in water (HFCS group) for 16 weeks. The effects of HFCS were assessed via enzyme-linked immunosorbent assays, histopathological assays of colon and bone, and 16S rDNA sequence analysis of gut microbiota. The serum of HFCS group mice had lower levels of bone alkaline phosphatase (BALP), bone Gla protein (BGP), insulin-like growth factor 1 (IGF-1), and testosterone, and higher levels of type I collagen carboxyl-terminal telopeptide (ICTP) and tartrate-resistant acid phosphatase (TRAP) than that of the control group. HFCS caused trabecular bone damage by decreasing trabecular number and thickness and increasing trabecular separation. The HFCS group colons were shorter than the control group colons. The HFCS-fed mice showed mild, localized shedding of epithelial cells in the mucosal layer, focal lymphocytic infiltration of the lamina propria, mild submucosal edema, and loosely arranged connective tissue. The HFCS group displayed lower abundance and altered composition of gut microbiota. The abundance of *Defluviitaleaceae UCG-011*, *Erysipelatoclostridium*, *Ruminococcaceae UCG-009*, *Lactobacillus*, *Blautia*, and *Parasutterella* increased, positively correlating with BALP, BGP, IGF-1, and testosterone levels, and negatively correlating with ICTP and TRAP levels. Our study revealed a potential diet-gut microbiota-bone health axis.

## Introduction

Osteoporosis is a systemic bone disease characterized by decreased bone density and bone quality, loss of bone microstructure, and increased bone fragility due to genetic and environmental factors. Currently, there are approximately 200 and 83.9 million patients with osteoporosis worldwide and in China, respectively. Reportedly, the treatment burden of osteoporosis and osteoporotic fractures has been rising rapidly. It is predicted that there will be 5.99 million patients with osteoporotic fractures in China by 2050 with a corresponding medical expenditure of up to 174.5 billion Chinese yuan (1).

In the absence of a cure, attention has been paid to the prevention of osteoporosis, and maximizing the peak bone mass (PBM) during bone formation represents an important preventive measure against osteoporosis. PBM is the maximal attained bone mass at the end of the growth period and is an important predictive indicator for the risk of osteoporosis and fractures that may occur in the future. In general, it is believed that the bone mass of males and females increases significantly in the first 20 years and reaches a plateau in late adolescence or early adulthood. The rate of bone formation exceeds that of bone resorption, leading to increased bone mass during childhood and adolescence, after which, the rate of bone resorption exceeds that of bone formation, resulting in constant bone loss (2). Therefore, it is important to determine factors leading to the increase in PBM to reduce the risk of osteoporosis. The determinants of PBM include genetic and lifestyle factors (i.e., exercise and diet). Given the feasibility of dietary changes, osteoporosis can be prevented by understanding dietary choices that affect bone health. Sugar intake is one of the dietary factors that may affect bone development as there has been an increasing number of studies showing that regular excessive intake of sugary foods negatively affects bone health (mass, morphology, microstructure, and mineralization of bone) (3).

There have been notable changes to the human diet over the past 20 years, with a tremendous increase in sugar intake. Many foods, such as beverages, biscuits or bread-like processed foods, flavored fermented milk, and chocolate products, have high sugar content. Caloric sweeteners include sugar (sucrose), high-fructose corn syrup (HFCS), honey, molasses, and fruit juice concentrates. At present, the most commonly used sweeteners are refined sugars and HFCS, which account, respectively, for 45 and 42% of caloric sweeteners used in United States food supplies (4). HFCS production in China has grown rapidly over the past decade with a 128.6% increase from 1.4 million tons in 2011/2012 to 3.2 million tons in 2018/2019 (5).

High-fructose corn syrup, also known as isomerized sugar, is a mixture of glucose and fructose. There are three major varieties of HFCS (namely HFCS-42, HFCS-55, and HFCS-90), among which, HFCS-55 is mainly used in the food industry (6).

In recent years, there has been an increasing number of studies investigating the effects of dietary intake of sugar on bone health, i.e., bone metabolism, bone histomorphometry, bone mineral content (BMC), bone mineral density, and mechanical strength. Gerbaix et al. (7) found that a high-fat/high-sucrose (HF/HS) diet improves bone density and bone mass, but does not change the tibial and vertebral trabecular bone volumes. Bass et al. (8) observed the effects of dietary fructose and glucose on bone formation, microstructure, and strength in animal models, and the results showed that the bone volume fraction (BV/TV) of the distal femurs was higher in fructose-fed rats than that in glucose-fed rats. In addition, the fructose-fed rats had a greater trabecular thickness and exhibited significantly higher maximum loads. Rats fed a high-fructose diet for 12 weeks displayed higher bone mass and better microstructure than those fed a high-glucose diet. The results reported by Yarrow et al. (9) showed that a high-fructose diet negatively affects the bones of 8-week-old male rats.

Besides, numerous studies have reported that a high-sugar diet can alter the gut microbiota and induce microbiota-mediated side effects in hosts. For instance, Do et al. (10) provided different types of diets (normal, high-sugar, and high-fructose) to 6-week-old C57BL/6J mice for 12 weeks, and found that the mice fed high-sugar and high-fructose diets had lower microbial diversity (i.e., fewer operational taxonomic units and lower Shannon index), lower abundance of Bacteroidetes, and higher abundance of Proteobacteria than that were fed a normal diet. The changes in abundance of these microbiotas were accompanied by a significant increase in intestinal permeability.

Hence, a better understanding of the effects of sugar intake on bones will facilitate the establishment of dietary recommendations for reducing the risk of osteoporosis. This study aimed to determine the effects of HFCS intake on the levels of serum bone metabolic markers, the number of femoral osteoclasts, trabecular bone parameters, and gut microbiota structure in growing male mice, as well as to observe the associations between HFCS intake, gut microbiota, and bone health.

## Results

### Effects of High-Fructose Corn Syrup on the Levels of Bone Metabolic Markers in the Serum of Growing Male Mice

**FIGURE 1:**
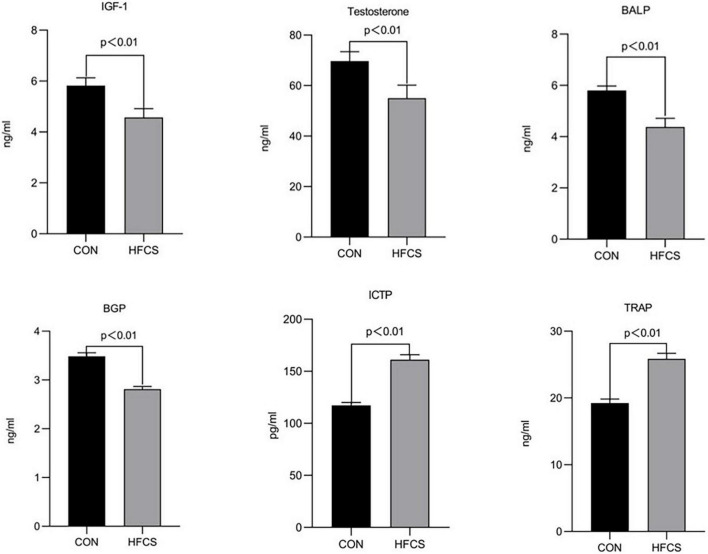
Effect of HFCS on serum BALP, BGP, ICTP, TRAP, IGF-1, and testosterone levels in mice. CON, control; HFCS, high-fructose corn syrup; BALP, bone alkaline phosphatase; BGP, bone Gla protein (osteocalcin); ICTP, type I collagen carboxyl-terminal telopeptide; TRAP, tartrate-resistant acid phosphatase; IGF-1, insulin-like growth factor 1. All data was expressed as mean ± SEM (*n* = 8) and analyzed by one-way ANOVA analysis.

After 16 weeks of continuous feeding, the mean serum BALP and BGP concentrations were approximately 5.8 and 3.5 ng/mL, respectively, in the HFCS group and 4.4 and 2.8 ng/mL, respectively, in the control group. The serum levels of BALP and BGP were significantly lower in the HFCS group than those in the control group (*p* < 0.01), indicating that HFCS inhibited bone formation in growing mice. Conversely, the mean serum ICTP and TRAP concentrations were approximately 117.3 pg/mL and 19.2 ng/mL, respectively, in the HFCS group and 161.1 g/mL and 25.9 ng/mL, respectively, in the control group. The levels of ICTP and TRAP were significantly higher in the HFCS group than in the control group (*p* < 0.01), indicating that HFCS promoted bone resorption in growing mice. In addition, the levels of testosterone and IGF-1 were significantly lower in the HFCS-fed mice than in the control mice (*p* < 0.01). These results are shown in Figure 1.

**FIGURE 2:**
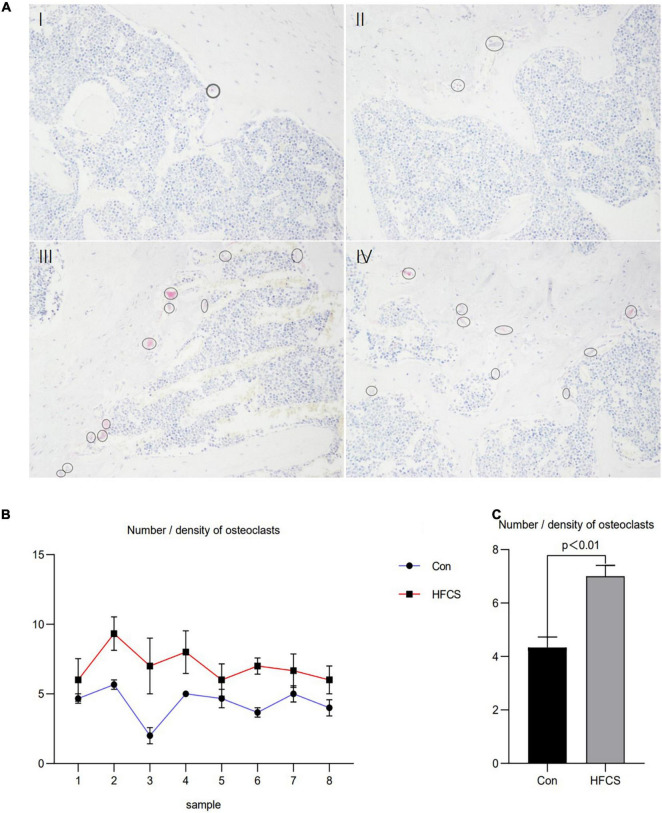
Effect of HFCS on the number and density of osteoclasts in male mice. Results of TRAP staining of mouse femurs showing in **(A)**, control (I, II) and HFCS (III, IV) groups. Image magnification 200 times, the circles and ellipses showing are osteoclasts. Plot of the number/density of osteoclasts in the femurs of the eight mice **(B)**. The average number/density of osteoclasts in the femurs of mice **(C)**. Data was expressed as mean ± SEM (*n* = 8) and analyzed by one-way ANOVA analysis.

Moreover, the number of osteoclasts in the femurs of the eight mice in the HFCS group was significantly higher than that in the control group. Next, the average number of osteoclasts in the femurs of the HFCS and control groups was compared, and this number was found to be significantly higher in the HFCS group than in the control group (*p* < 0.05) as determined by TRAP staining. The changes in the number and density of osteoclasts are shown in Figure 2.

**FIGURE 3:**
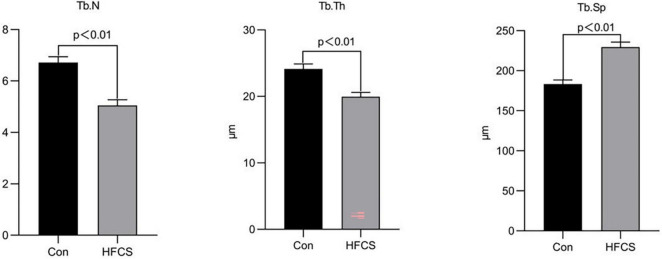
Effects of HFCS on the Tb.N, Tb.Th, and Tb.Sp in mice. Tb.N, trabecular number per mm; Tb.Th, trabecular thickness; Tb.Sp, trabecular separation. All data was expressed as mean ± SEM (*n* = 8) and analyzed by one-way ANOVA analysis.

The mean trabecular number per mm (Tb.N) and trabecular thickness in μm (Tb.Th) of mice in the HFCS group were approximately 5.05 and 19.96 μm, respectively, whereas those of the control group were 6.72 and 24.13 μm, respectively. The Tb.N and Tb.Th of mice in the HFCS group were significantly lower than those in the control group (*p* < 0.01). Tb.Sp was higher in the HFCS group than in the control group (*p* < 0.01). Tb.Th and Tb.N were used to characterize changes in bone mass. When Tb.N was held constant, a higher Tb.Th indicated greater bone mass. Similarly, when Tb.Th was held constant, a higher Tb.N indicated greater bone mass. For Tb.Sp, greater trabecular spacing indicates greater severity of osteoporosis. These data showed that HFCS was harmful to the trabecular bone of growing mice (Figure 3).

### Effects of High-Fructose Corn Syrup on the Colonic Length and Colon Pathology of Growing Male Mice

**FIGURE 4:**
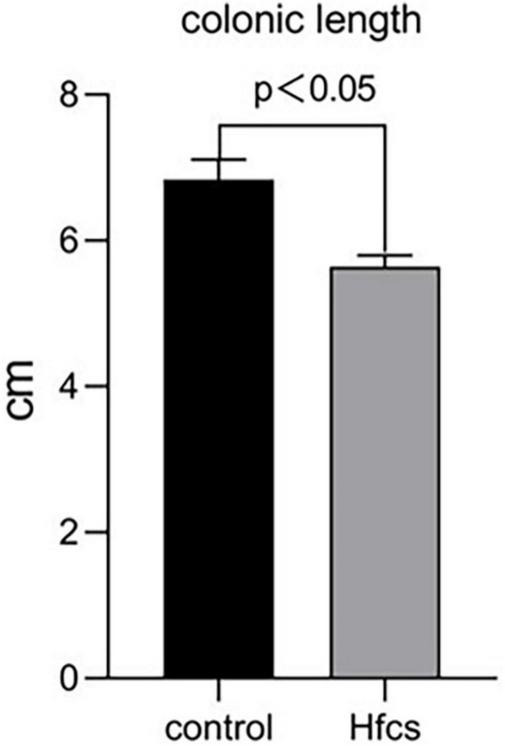
Effect of HFCS on colonic length in mice. Data was expressed as mean ± SEM (*n* = 8) and analyzed by one-way ANOVA analysis.

**FIGURE 5:**
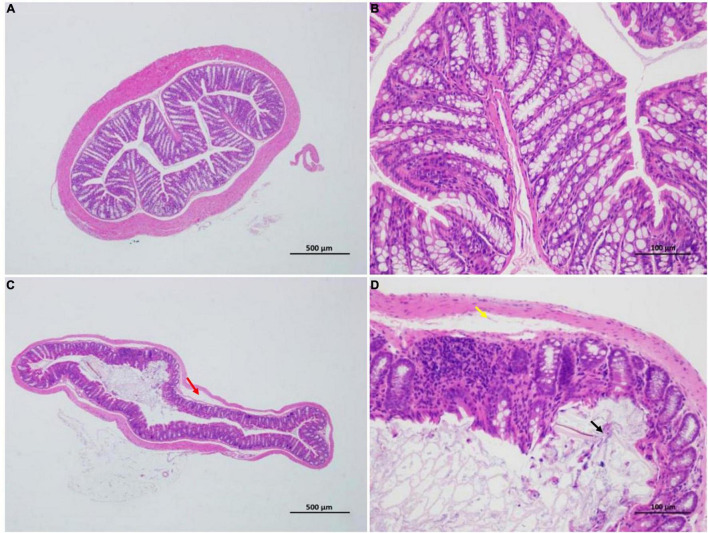
Effect of HFCS on the colon pathology of mice (*n* = 8). Control group **(A,B)**; distinct structure of circular folds, intact intestinal epithelium (simple columnar epithelium) in the mucosal layer, epithelial cells with a normal morphological structure, abundant intestinal glands in lamina propria, relatively high number of goblet cells, uniformly stained muscular layer, orderly arranged muscular fibers with normal morphological structure, and no apparent signs of inflammation. In summary, the control group was rated 0 **(A,B)**. HFCS group **(C,D)**; minor localized shedding of epithelial cells, massive epithelial lifting down the sides of villi (black arrow) in the mucosal layer, localized focal lymphocytic infiltration, sections with extension of the subepithelial space and moderate lifting of the epithelial layer from the lamina propria (yellow arrow) in the lamina propria, mild localized edema in the submucosa, and loosely arranged connective tissues (red arrow). Therefore, HFCS group **(C,D)** received a score of 3.

Mice in the HFCS group had significantly shorter colons than mice in the control group (*p* < 0.05) (Figure 4). The former mice also manifested signs of inflammation after being fed continuously with HFCS for 16 weeks (Figure 5).

### Effects of High-Fructose Corn Syrup on the Gut Microbiota in Growing Male Mice

**FIGURE 6:**
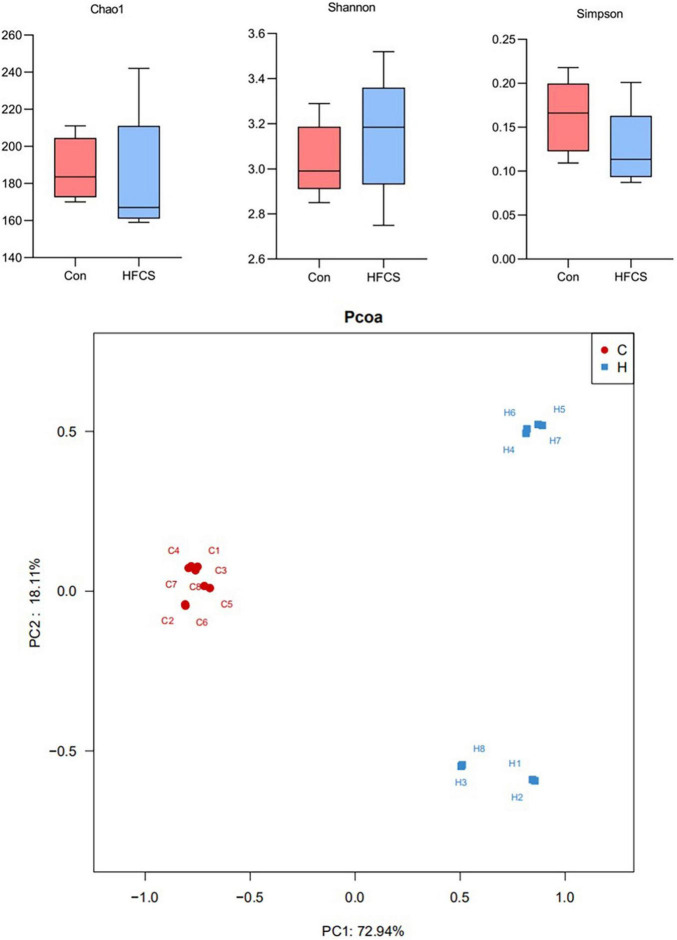
Schematic diagram of alpha and beta diversity analyses. C, control; H, high-fructose corn syrup; PC, principal component (*n* = 8).

Upon 16S rRNA gene sequencing, the resulting sequences were subjected to alpha diversity analysis. There was a statistically significant difference in the Chao1 index (*p* < 0.05), but no statistically significant difference was observed in the Shannon and Simpson indices between the control and HFCS groups (*p* > 0.05) (Figure 6). The Chao1 index of the HFCS group was significantly lower than that of the control group, suggesting that HFCS reduced the abundance of the gut microbiota components in mice.

The principal component analysis (PCoA) plot (Figure 6) shows the difference in the microbial structure of colon samples from the control and HFCS groups. Closer distances between points in the PCoA plot indicate more similarity in microbial structure in the samples. Figure 6 shows a large distance between the colon samples of the HFCS group and the control group, indicating that HFCS causes some change in the microbial structure of the mouse gut.

**FIGURE 7:**
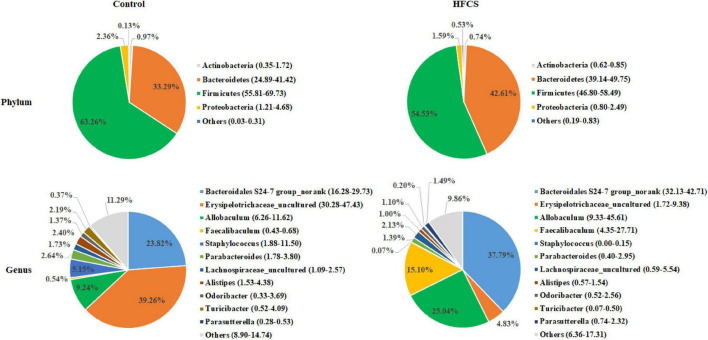
Phylum- and genus-level pie charts of the structure of the intestinal microflora in control and HFCS-fed mice (n = 8).

The analysis of phylum-level differences in gut microbiota showed that mice in the HFCS group had a lower abundance of Firmicutes and Actinobacteria, a significantly lower abundance of Proteobacteria, and a higher abundance of Bacteroidetes than the mice in the control group (Figure 7). The analysis of genus-level differences revealed that the HFCS group mice had a higher abundance of *Bacteroidales S24-7 group_norank*, *Allobaculum*, and *Faecalibaculum*, as well as a lower abundance of *Erysipelotrichaceae_uncultured* and *Staphylococcus* than the control group mice (Figure 7).

**FIGURE 8:**
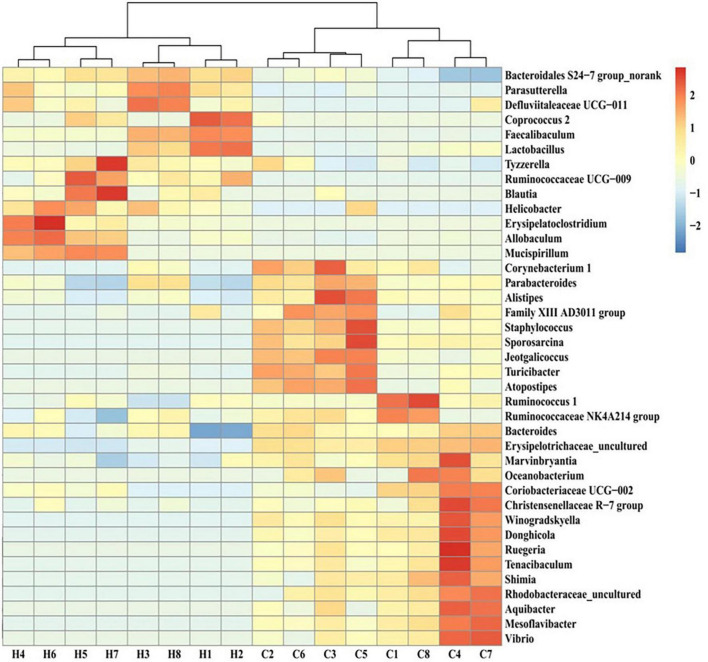
Heat map of the intestinal flora of different genera in control and HFCS-fed mice. C1–C8, individual control mice; H1–H8, individual HFCS-fed mice.

The screening for bacterial genera with abundance levels of ≥0.01 and statistically significant differences in the abundance of gut microbiota members between the HFCS and control groups (*p* < 0.05) revealed that the abundance of *Bacteroidales S24-7 group_norank*, *Parasutterella*, *Ruminococcaceae UCG-009*, *Defluviitaleaceae UCG-011*, *Coprococcus 2*, *Faecalibaculum*, *Lactobacillus*, *Tyzzerella*, *Blautia*, *Helicobacter*, *Erysipelatoclostridium*, *Allobaculum*, and *Mucispirillum* increased in the HFCS group, whereas that of *Winogradskyella*, *Sporosarcina*, *Donghicola*, *Rhodobacteraceae_uncultured*, *Mesoflavibacter*, *Shimia*, *Marvinbryantia*, *Staphylococcus*, *Turicibacter*, *Tenacibaculum*, *Ruegeria*, *Alistipes*, *Jeotgalicoccus*, *Aquibacter*, *Vibrio*, *Corynebacterium 1*, *Oceanobacterium*, *Bacteroides*, *Atopostipes*, *Christensenellaceae R-7 group*, *Ruminococcaceae NK4A214 group*, *Family XIII AD3011 group*, *Parabacteroides*, *Coriobacteriaceae UCG-002*, *Erysipelotrichaceae_uncultured*, and *Ruminococcus 1* decreased (Figure 8).

### Correlation Analysis Between Bone Metabolism Markers and Differentially Abundant Bacterial Genera

**FIGURE 9:**
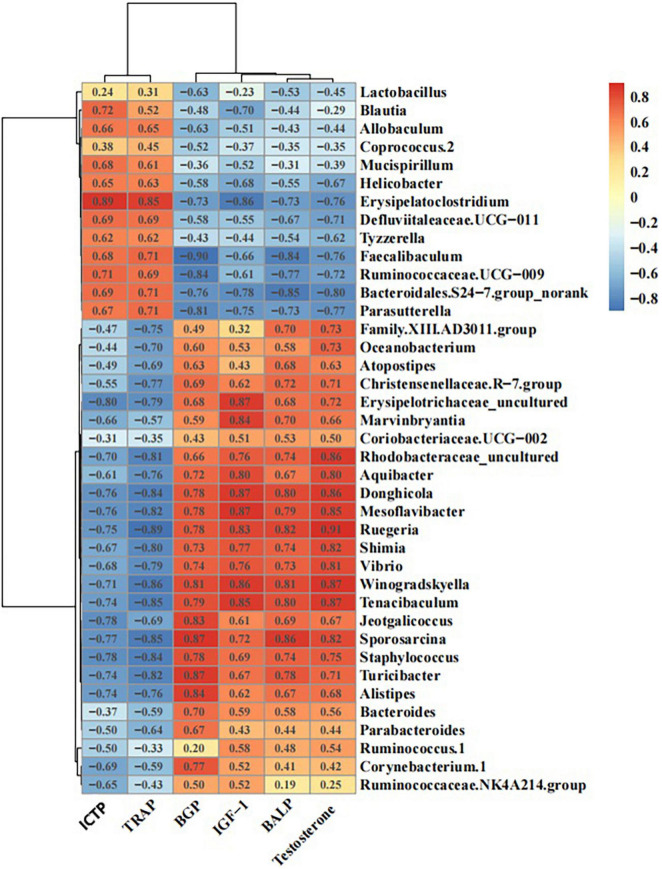
Schematic diagram of the correlations between bone metabolic indices and intestinal flora.

The correlation analysis between the serum levels of BALP, BGP, ICTP, TRAP, IGF-1, or testosterone and the abundance of different gut microbiota members in mice showed that the abundance of *Defluviitaleaceae UCG-011*, *Erysipelatoclostridium*, *Ruminococcaceae UCG-009*, *Blautia*, and *Parasutterella* had significant negative correlations with the levels of BALP, BGP, IGF-1, and testosterone, as well as significant positive correlations with the levels of ICTP and TRAP. In contrast, the abundance of *Rhodobacteraceae_uncultured*, *Winogradskyella*, *Donghicola*, *Ruminococcus 1*, and *Mesoflavibacter* displayed significant positive correlations with the levels of BALP, BGP, IGF-1, and testosterone, as well as significant negative correlations with the levels of ICTP and TRAP (Figure 9).

## Discussion

Our study revealed that HFCS reduced the levels of bone formation markers (BALP and BGP), IGF-1, and testosterone, and increased the levels of bone resorption markers (ICTP and TRAP) in the serum of mice, as well as the number of osteoclasts in the femurs of the mice. HFCS also caused damage to the trabecular bone with decreases in the Tb.N and Tb.Th, as well as an increase in the Tb.Sp.

The health impacts of frequent excessive intake of HFCS-containing foods have been demonstrated in previous studies. High intake of HFCS is related to increased risk of obesity, diabetes, cardiovascular diseases, and metabolic syndrome. Consumption of beverages containing HFCS has also been associated with the risk of non-alcoholic fatty liver disease and gout (in men) (11). Moreover, the effects of HFCS on bone health have been reported. Tsanzi et al. (12) found that a high-HFCS-55 diet reduced the relative weight and BMC of femurs in rats.

Bone remodeling is a lifelong process, in which mature bone tissue is absorbed and new bone tissue is synthesized. This process is regulated by various cell types, including osteoblasts (bone formation), osteocytes (bone homeostasis), osteoclasts (bone resorption), and immune cells (bone resorption and formation). The rate of bone formation exceeds that of bone resorption, leading to increased bone mass during childhood and adolescence. A disrupted balance between bone formation and bone resorption, i.e., when the rate of bone resorption exceeds that of bone formation, will result in decreased bone mass and osteoporosis (13).

Bone turnover biomarkers (BTMs) are by-products of bone remodeling and their urinary or serum levels can be measured to determine the rate of bone turnover (14). BTMs are further categorized into bone formation markers [e.g., BALP, N-terminal propeptide of type 1 collagen (P1NP), and BGP] and bone resorption markers [e.g., TRAP, ICTP, C-telopeptide of type I collagen (CTX-1), and n-telopeptide of type 1 collagen (NTX-1)] (15). Unlike bone density, BTM levels are clinically useful as they change rapidly in response to changes in the bone turnover rate and display a clear representation of these changes for clinical use. Hence, they can be applied as diagnostic tools for patients lacking any manifestation of osteoporosis or when imaging data are unavailable. Testosterone plays an important role in bone growth and metabolism, maintenance of bone mass, and resistance to bone loss. Testosterone plays particularly prominent roles during childhood, such as enabling the thickening and growth of bones by promoting the development of skeletal muscles and calcification of bones. During adolescence, testosterone primarily plays an important role in reaching PBM by improving the bone mass of cancellous and cortical bones. After reaching adulthood, testosterone mainly promotes bone formation and inhibits bone resorption, acting jointly with other hormones that regulate bone metabolism to maintain bone mass and regulate bone metabolism (16).

Here, we uncovered that HFCS can alter the biodiversity of the gut microbiota in mice, whereby it reduced the abundance of Firmicutes, Proteobacteria, and Actinobacteria, and increased that of Bacteroidetes at the phylum level. We also identified 39 bacterial genera that showed differential changes in abundance, of which 13 showed increased abundance (*Bacteroidales S24-7 group_norank*, *Parasutterella*, *Ruminococcaceae UCG-009*, *Defluviitaleaceae UCG-011*, *Coprococcus 2*, *Faecalibaculum*, *Lactobacillus*, *Tyzzerella*, *Blautia*, *Helicobacter*, *Erysipelatoclostridium*, *Allobaculum*, and *Mucispirillum*), while the remaining 26 showed reduced abundance. These observations are consistent with the findings of previous studies. The guts of mice fed a high-sugar or high-fructose diet contained a relatively lower proportion of *Bacteroides* and a significantly higher proportion of *Proteus* (10). Another study, which explored the effects of four different diets given in succession on the gut microbiota in humans, revealed that HFCS intake induced significant changes in the composition of microbiota with a lower abundance of Firmicutes and *Ruminococcus*, and a higher abundance of Bacteroidetes compared to that induced by a fruit-rich diet (17). HFCS intake also reduced the number of *Faecalibaculum* and *Staphylococcus* compared to that induced by the low-fructose diet (17). The proportion of lactic acid bacteria generally decreases in mice fed normal or glucose-containing foods within 10 weeks but shows an opposite trend in fructose-fed mice (18). Interestingly, we also observed an increase in the abundance of *Lactobacillus* in the colons of HFCS-fed mice, probably due to different proportions of fructose and glucose in HFCS or different metabolic rates of fructose and glucose in the gastrointestinal tract of mice.

*Defluviitaleaceae UCG-011*, *Erysipelatoclostridium*, *Ruminococcaceae UCG-009*, *Lactobacillus*, *Blautia* (which belong to the phylum Firmicutes), and *Parasutterella* (which belongs to the phylum Proteobacteria) have previously been demonstrated to exhibit pro-inflammatory properties. Zha et al. (19), Sun et al. (20), and Wang et al. (21) found that the relative levels of these bacterial genera increased in mice with dextran sulfate sodium (DSS)-induced colitis and were restored to normal levels following the alleviation of colitis. Balish and Warner (22) found that the inoculation of *Enterococcus faecalis* causes chronic, progressive inflammatory bowel disease in germ-free IL-10-knockout mice. Similarly, *Ruminococcus* strains, which displayed significant correlations with Crohn’s disease (CD), were isolated from the small intestinal mucosa of patients with CD via anaerobic cultivation and inoculated into germ-free mice, promoting the recruitment of TH1 and TH17 cells into the mouse intestines (23). The above studies have confirmed that these members of the gut microbiota exhibit pro-inflammatory properties and play important roles in the onset and persistence of colitis.

Gut microbiota has an indisputable influence on bone health. A study by Sjogren et al. (24) revealed the connection between gut microbiota and bone health. The use of probiotic supplements (*Bifidobacterium* and *Lactobacillus*) and the potential benefits of prebiotics on bone health under both healthy and pathological conditions have confirmed the major role of the gut microbiome in regulating bone health (25–27). It is currently believed that the gut microbiome affects bone metabolism through several pathways (28), among which the most studied is the one where the gut microbiota activate local and systemic immune/inflammatory responses to produce circulating cytokines that have major effects on bone metabolism. In addition, the gut microbiome acts as a virtual endocrine organ of the human body, interacting with the endocrine system to regulate circulating IGF-1 levels, thereby affecting bone homeostasis (29).

Here, we found that HFCS-fed mice had shorter, inflamed [as indicated using hematoxylin and eosin (HE) staining] colons. We also observed increases in the abundance of bacteria with pro-inflammatory properties at the genus level (e.g., *Defluviitaleaceae UCG-011*, *Erysipelatoclostridium*, *Ruminococcaceae UCG-009*, *Coprococcus 2*, *Blautia*, *Allobaculum*, *Parasutterella*, and *Lactobacillus*). Additionally, the abundances of these gut microbiota members were significantly and positively correlated with the levels of bone resorption markers and negatively correlated with the levels of bone formation markers in the serum of mice. Moreover, mice in the HFCS group showed reduced serum levels of IGF-1. These results suggest that HFCS intake affects bone health by regulating the structure of the gut microbiota.

The results of the present study show that dietary HFCS inhibits osteogenesis, promotes bone resorption, and damages trabecular bone in growing mice. This damage may be caused by gut microbiome regulation that induces local colon or systemic inflammation, which changes the levels of cytokines that affect bone metabolism. This indicates the possible existence of a diet-gut microbiome-bone health axis.

## Materials and Methods

### Animals

Sixteen 3-week-old clean male mice (weighing between 18 and 22 g) were purchased from Shanghai SLAC Laboratory Animal Co., Ltd. [License No. SCXK(Lu)2017-0005]. The study was carried out at the Laboratory Animal Center of Zhejiang Academy of Agricultural Sciences (Animal Experimentation License No. 286868667) and was approved by the Zhejiang Provincial Ethics Committee for Laboratory Animals (Ethical Approval No. 78865576). After 1 week of adaptive feeding, the mice were randomly divided into control (Con) and HFCS groups. The mice in both groups were fed standard chow and provided either purified water alone (for the Con group) or purified water containing 30% HFCS (for the HFCS group). All animals were kept in the same clean room in groups of four mice per cage at a temperature of 24–28°C and relative humidity of 80% under a 12:12-h light/dark cycle with one bedding change every 3 days. The mice were given *ad libitum* access to water and food (standard animal feed provided by the Laboratory Animal Center). After 16 weeks, the mice were anesthetized and euthanized via exsanguination of the abdominal aorta to harvest colons, colonic contents, and left femurs.

### Serum Collection and Detection of Related Markers

Serum was extracted from collected blood samples via centrifugation at 4,000 × *g* for 10 min at 4°C and then stored in a freezer at −20°C. Enzyme-linked immunosorbent assay of serum osteocalcin (BGP), bone alkaline phosphatase (BALP), type I collagen carboxyl-terminal peptide (ICTP), tartrate-resistant acid phosphatase (TRAP), testosterone, and insulin-like growth factor (IGF)-1 levels was performed by Servicebio Technology Co., Ltd. (Wuhan, China). Briefly, plates and strips were removed from the aluminum foil bag after equilibrating for 1 h at 20°C. Standard and sample wells were set up, and 50 μL of different concentrations of standards was added to each standard well. Then, 50 μL of each sample to be tested was added to each sample well; no sample was added to the blank wells. Thereafter, 50 μL of biotin-labeled antibody was added to each well, followed by sealing of the wells with a membrane, and incubated in a 37°C incubator for 30 min; no antibody was added to the standard and blank wells. After discarding the supernatant, the plate was dried with absorbent paper and washed five times. Except for the blank wells, 100 μL of horseradish peroxidase (HRP)-labeled detection antibody was added to each standard and sample well, the wells were sealed with a membrane, and incubated in a 37°C incubator for 30 min. The supernatant was again discarded, and the plate was dried with absorbent paper and washed five times. Then, 50 μL of substrates A and B was added to each well, and the plate was incubated at 37°C for 15 min in the dark. Fifty microliters of stop solution was then added to each well, and the absorbance of each well was measured within 15 min at a wavelength of 450 nm, and the concentration of the assayed molecule was calculated.

### Preparation of Bone Specimens and Measurement of Relevant Indicators

Femur samples were fixed in 4% paraformaldehyde for 2 days. Then they were trimmed, dehydrated, embedded, and sectioned in strict accordance with the standard operating procedures of the Servicebio Technology Co., Ltd., pathology laboratory.

The bone tissue sections were removing paraffin wax, followed by staining with TRAP dye liquor and staining with hematoxylin before embedding in neutral resin. Finally, the tissue sections were embedded in neutral resin and examined under an Eclipse Ci-L microscope equipped with a camera for image acquisition and analysis of target tissue areas at 200× magnification. The resulting microscopic images were used to determine the number of osteoclasts in three fields of view per section and the corresponding tissue area (mm^2^) using Image-Pro Plus 6.0 software. The osteoclast density was then calculated as follows: osteoclast density (n/mm^2^) = number of osteoclasts/tissue area.

The bone tissue sections were removing paraffin wax, followed by staining with Safranin O, destaining, and staining with Fast Green before embedding in neutral resin. After that, selected target areas of bone tissues were imaged at 20 × magnification using the Eclipse Ci-L microscope equipped with a camera and analyzed using Image-Pro Plus 6.0 software. Millimeters were the standard unit for the measurement of total cancellous bone area (T.Ar), trabecular area (Tb.Ar), and trabecular perimeter (Tb.Pm). The following values were then calculated:

Percentage area of trabecular bone (%Tb.Ar) = Tb.Ar/T.Ar*100

Trabecular thickness in μm (Tb.Th) = (2000/1.199)* (Tb.Ar/Tb.Pm)

Trabecular number per mm (Tb.N) = (1.199/2)* (Tb.Pm/Tb.Ar)

Trabecular separation in μm (Tb.Sp) = (2000/1.19)/1.19)* (T.Ar−Tb.Ar)/Tb.Pm

After exposing the abdominal cavity using scissors, the colon was carefully separated and gently straightened to measure its length with a measuring tape. Statistical analyses were performed by SPSS 23.0 (IBM, New York, NY, United States) using One way ANOVA.

The colonic samples of mice were fixed with 4% paraformaldehyde, paraffin embedded, and sectioned for HE staining by Servicebio Technology Co., Ltd., to observe histopathological changes under a high-power microscope named Nikon Eclipse E100 and it’s imaging system Nikon DS-U3. Morphologic changes were assessed and graded on a scale of 0–5 using the intestinal injury score developed by Chiu et al. (30). Based on mucosal changes, the grades were assessed between 0 and 5 as follows: Grade 0, normal mucosal villi; grade 1, subepithelial space can be seen at the apex of the villus; grade 2, sections with extension of the subepithelial space and moderate lifting of the epithelial layer from the lamina propria; grade 3, massive epithelial lifting down the sides of villi; grade 4, sections with denuded villi and dilated capillaries; and grade 5, sections with digestion and disintegration of lamina propria.

### Collection, Processing, and 16S rDNA Sequencing of Mouse Colonic Contents

The colonic contents were collected and temporarily stored at −80°C for subsequent 16S rDNA sequencing by Novogene (Beijing, China). Genomic DNA extracted from the colonic samples was subjected to electrophoresis in 1% agarose gels. Barcoded specific primers (515F: 5′-GTGCCAGCMGCCGCGG-3′; 907R: 5′-CCGTCAATTCMTTTRAGTTT-3′) were synthesized to amplify the V3 + V4 region of the 16S rDNA gene. After being separated via electrophoresis, the resulting bands were excised to recover the amplicons, which were then quantified using a QuantiFluor™ Fluorometer and sequenced using the HiSeq 2500 System to generate 250-bp paired-end reads (PE250). Effective sequences of all samples need to be obtained according to barcode. Then the quality of reads was controlled and filtered. Then, according to overlap relation between PE reads, the paired reads are merged into a sequence. Finally, the high-quality sequence of each sample was obtained by splitting the barcode and primer sequences, and the sequence direction was corrected and the chimera was removed according to the positive and negative barcode and primer directions during the process. The resulting effective tags were subjected to operational taxonomic unit analysis, species classification, alpha diversity analysis, and beta diversity analysis to investigate the composition and changes in the gut microbiota.

### Statistical Analysis

All data were analyzed using SPSS 23.0 software and expressed as the mean ± standard deviation. Comparisons among multiple groups were carried out using one-way ANOVA and tested for homogeneity of variance. Multiple comparisons of mean values between groups were carried out using the Student–Newman–Keuls method. Operational taxonomic units (OTUs) were defined as the minimum of 97% sequence similarity. bacterial abundance was estimated using Chao1 and the number of observed species diversity was analyzed using Shannon. PCOA statistical analysis and mapping were performed with R language. Correlation analysis of environmental factors and bacterial abundance was carried out by using ReDundancy Analysis (RDA) and Pearson’s Correlation coefficient. *P* < 0.05 indicated statistically significant differences. *P* < 0.01 indicated highly statistically significant differences.

## Data Availability Statement

The raw data supporting the conclusions of this article will be made available by the authors, without undue reservation.

## Ethics Statement

The animal study was reviewed and approved by the Zhejiang Provincial Ethics Committee for Laboratory Animals.

## Author Contributions

ZF, YC, XL, and XW: data curation. HS and JL: funding acquisition. ZF, YC, and LZ: investigation. XH and JL: project administration. HS: resources. XH: writing – original draft. JL: writing – review and editing. All authors contributed to the article and approved the submitted version.

## Conflict of Interest

The authors declare that the research was conducted in the absence of any commercial or financial relationships that could be construed as a potential conflict of interest.

## Publisher’s Note

All claims expressed in this article are solely those of the authors and do not necessarily represent those of their affiliated organizations, or those of the publisher, the editors and the reviewers. Any product that may be evaluated in this article, or claim that may be made by its manufacturer, is not guaranteed or endorsed by the publisher.

## Abbreviations

BALP: bone alkaline phosphatase
BGP: bone Gla protein
BMC: bone mineral content
CD: Crohn’s disease
HE: hematoxylin and eosin
HFCS: high-fructose corn syrup
ICTP: I collagen carboxyl-terminal peptide
PBM: peak bone mass
PC: principal component
PCoA: principal component analysis
TRAP: tartrate-resistant acid phosphatase.
